# High colloidal stability ZnO nanoparticles independent on solvent polarity and their application in polymer solar cells

**DOI:** 10.1038/s41598-020-75070-0

**Published:** 2020-10-22

**Authors:** Woojin Lee, Jiwoo Yeop, Jungwoo Heo, Yung Jin Yoon, Song Yi Park, Jaeki Jeong, Yun Seop Shin, Jae Won Kim, Na Gyeong An, Dong Suk Kim, Jongnam Park, Jin Young Kim

**Affiliations:** 1 Department of Energy Engineering, Ulsan National Institute of Science and Technology (UNIST), 50 UNIST-gil, Ulju-gun, Ulsan, 44919 Republic of Korea; 2 Department of Physics, Ulsan National Institute of Science and Technology (UNIST), 50 UNIST-gil, Ulju-gun, Ulsan, 44919 Republic of Korea; 3 KIER-UNIST Advanced Center for Energy, Korea Institute of Energy Research (KIER), UNIST-Gil 50, Eonyang-eup, Ulju-gun, Ulsan, 689-851 Republic of Korea

**Keywords:** Materials for devices, Nanoscale materials, Energy science and technology

## Abstract

Significant aggregation between ZnO nanoparticles (ZnO NPs) dispersed in polar and nonpolar solvents hinders the formation of high quality thin film for the device application and impedes their excellent electron transporting ability. Herein a bifunctional coordination complex, titanium diisopropoxide bis(acetylacetonate) (Ti(acac)_2_) is employed as efficient stabilizer to improve colloidal stability of ZnO NPs. Acetylacetonate functionalized ZnO exhibited long-term stability and maintained its superior optical and electrical properties for months aging under ambient atmospheric condition. The functionalized ZnO NPs were then incorporated into polymer solar cells with conventional structure as n-type buffer layer. The devices exhibited nearly identical power conversion efficiency regardless of the use of fresh and old (2 months aged) NPs. Our approach provides a simple and efficient route to boost colloidal stability of ZnO NPs in both polar and nonpolar solvents, which could enable structure-independent intense studies for efficient organic and hybrid optoelectronic devices.

## Introduction

ZnO is known as a multifunctional material, which possesses high electrical conductivity^1^ and visible transparency (optical band gap, E_opt_ > 3.3 eV), photostability^2,3^, and photocatalytic ability^4,5^. In particular, it has been often utilized as a highly efficient n-type buffer layer of organic optoelectronic devices due to its proper energy level alignment to the active layer with ideal band dispersion^6^ for the selective charge extraction (or injection). Sol–gel and sputtering methods are being widely adopted for the preparation of ZnO thin film^7–11^. But the methods cause inevitable damages by thermal annealing or ion bombardments on underlying layers during the deposition processes. It forces their applications to optoelectronic devices only with inverted structure, which the underlying layer is robust oxide layer in most cases. Consequently, synthesis of ZnO nanoparticles (NPs) have been intensively studied as an alternative method, which are able to reduce processing temperature under 150 ℃^12,13^ or even requires no thermal annealing^14^ to form ZnO thin film. Most studies dispersed the NPs in nonpolar solvent with steric stabilization using long alkyl acid or amine ligands^15,16^, or dispersed in the mixture of polar and nonpolar solvents, which greatly improves colloidal stability compared to the stability of the NPs in mono-solvent^17,18^. The presence of nonpolar solvent in the solution, however, forces NPs to be used only in the inverted structure in the same manner as sol–gel or sputtering processed ZnO. Therefore, it is of great importance to improve colloidal stability of ZnO NPs in polar solvent to improve a versatility of the NPs in device applications. Methanol is one of the best candidates as an appropriate solvent for the NPs to form a thin film on organic layer with least damages, due to its solvent orthogonality and high volatility^19–21^. Li et al. reported methanol treatment on poly[[2,6′‐4,8‐di(5‐ethylhexylthienyl)benzo[1,2‐b;3,3‐b]dithiophene][3‐fluoro‐2[(2‐ethylhexyl)­carbonyl]thieno­[3,4‐b]thiophenediyl]] (PTB7-Th) : phenyl-C_71_-butyric acid methyl ester (PC_71_BM) blend could rearrange the blend morphology toward favorable morphology and achieved the enhanced power conversion efficiency (PCE) by 9.8%^19^. Guo et al. tested alcohol treatment on the active blend using various alcohols of methanol, ethanol, 2-propanol, and 1-butanol^20^. Among them, methanol efficiently reconstructed the inner structure of the blend and altered the energy level at the interfaces between the active layer and the top electrode for better charge extraction. Based on the previous studies, it is expected that ZnO NPs in methanol would possess the advantages of both materials; methanol reconstructs the molecular packing of the active layer toward better morphology and ZnO NPs act as an efficient electron transport layer in the device. Despite of the prescribed advantages, poor colloidal stability of ZnO NPs in methanol frustrates their applications in the organic optoelectronics with conventional structure and comprehensive studies on improving colloidal stability of ZnO NPs in methanol solvent have been hardly studied so far.

In this manuscript, we report the greatly improved colloidal stability of ZnO NPs in methanol by adding a coordination complex, titanium diisopropoxide bis(acetylacetonate) (Ti(acac)_2_), denoted as Ti(acac) in the following, as an efficient stabilizer. Metal acetylacetonates have been widely used as efficient metal precursor to synthesize metal and metal oxide nanoparticles. But few studies have provided systematic analysis on metal acetylacetonates as ligands for the nanoparticles. We found that Ti(acac) is readily dissociated in methanol and forms Zn-acac bonds at the surface of ZnO NPs and Ti element, from Ti(acac), exists as Ti^4+^ state in oxide lattice. Additional electrons provided by Ti atoms effectively fills up deep trap levels, which results in the reduction of green luminescence from the NPs. In addition, acac functionalized ZnO NPs (fZnOs) exhibit significantly reduced aggregation between NPs in methanol and show long-term (for months) colloidal stability not only in methanol, but also in isopropanol and even nonpolar solvent such as chlorobenzene. Superior colloidal stability of fZnOs is further proved in polymer solar cell (PSC) applications. PTB7-Th:PC_71_BM devices using 2 months-aged pristine ZnOs (pZnOs) results in significantly decreased PCE by 47.5%, compared to the devices with as-prepared pZnOs, which is attributed to the aggregation of the NPs. While, the devices with as-prepared and 2 months-aged fZnO exhibits nearly identical PCE. The results provide simple and efficient routes to improve colloidal stability of metal oxide nanoparticles and extend their applications to a variety of device structures of organic optoelectronic devices.

## Results

### Characterization of functionalized ZnO nanoparticles

For the preparation of stable ZnO NP solutions in methanol, 20.5 μM, 41.0 μM, and 82.0 μM of Ti(acac) was added to the pZnO NP solutions which were prepared by using previously reported method^22,23^. The detailed procedure can be found in **Method** section. In the following, each functionalized ZnO NP solution is denoted as fZnO-L, fZnO-M, and fZnO-H (low, mid, and high in concentration), respectively. ZnO NP solution without Ti(acac) was also studied as a reference (denoted as pZnO – pristine ZnO NPs).

**Figure 1:**
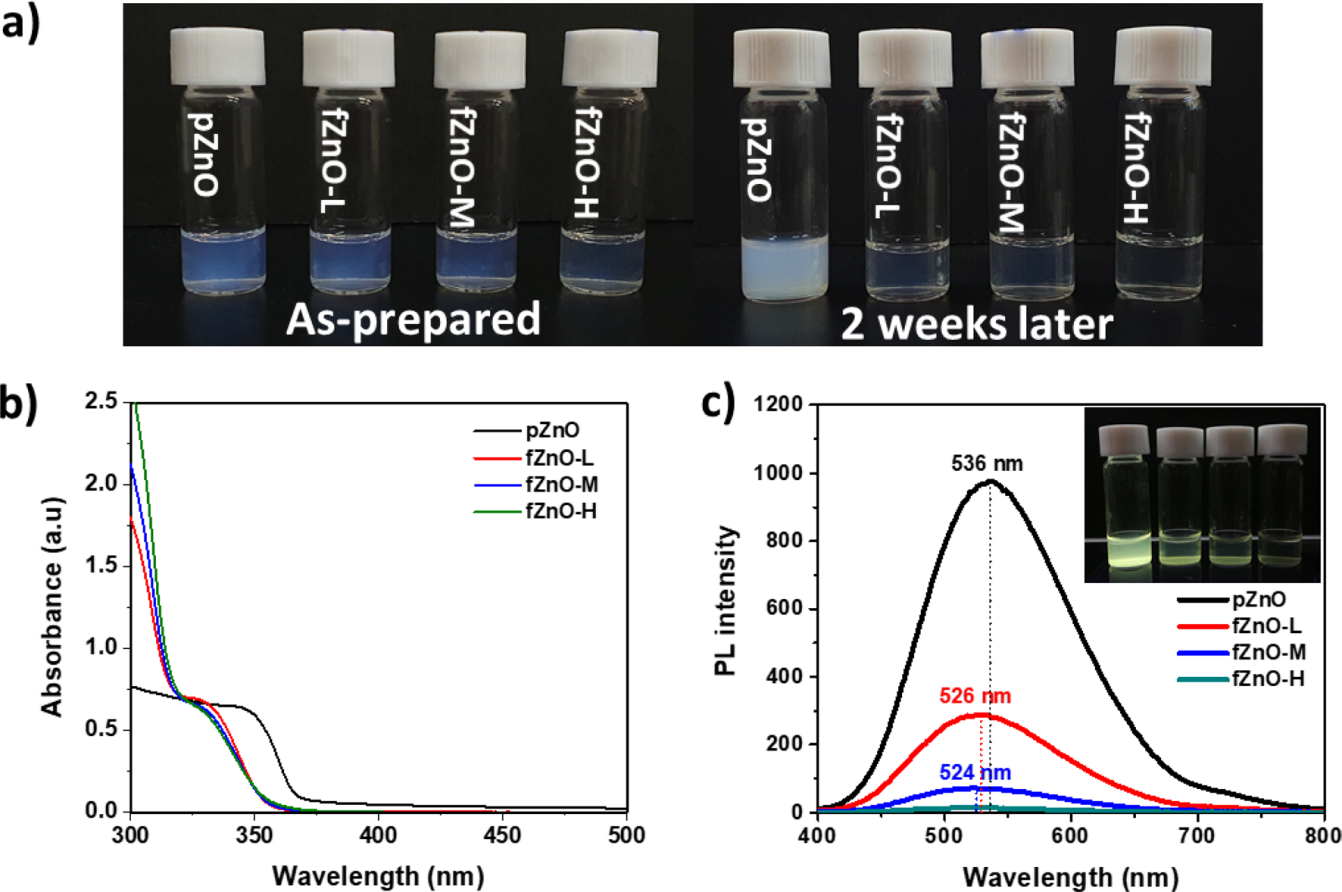
Stabilized ZnO NP solutions after the addition of Ti(acac). (**a**) Photographs of ZnO NP solutions dispersed in MeOH. (**b**) UV–Vis absorption and (**c**) photoluminescence spectra of ZnO NP solutions.

Figure 1a shows photographs of the prepared solutions with three different Ti(acac) concentrations. As-prepared pZnO was translucent and easily filtered via 0.45 μm pore-sized PTFE filter. An addition of Ti(acac) with three different concentration (low, mid, and high) made the solutions transparent. 2 weeks later, pZnO showed milky suspension, implying that strong aggregation occurred between NPs because of their high surface energy, while fZnO-L, M, and H maintained its high transparency. This observation suggests that the addition of Ti(acac) successfully prevents NPs from the aggregation in methanol solvent. Figure 1b exhibits the UV–Vis absorption spectra of the solutions with different Ti(acac) concentrations. Absorption onset of the reference solution was 368 nm, corresponding to 3.37 eV of optical bandgap. The addition of Ti(acac) led to blueshift of absorption onset to 355 nm (3.49 eV) for fZnO-L, and 353 nm (3.51 eV) for fZnO-M and H. The slight widening in bandgap is due to weak quantum confinement of ZnO nanoparticles, caused by a loss of bulk properties after the segregation, as well as reduced light scattering by NP lomerates^24^. Figure S1 in Supplementary Material shows that pZnO exhibits stronger light scattering over whole wavelengths as it is stored longer, while fZnOs maintain sharp absorption onset. Same tendency was observed in photoluminescence (PL) spectra in Fig. 1c. As increasing the concentration of Ti(acac), PL peak at 536 nm was blue-shifted to 524 nm with decreasing the peak intensity and finally disappeared in fZnO-H. Despite the fact that its exact origin is still under debates^25–28^, green luminescence (GL) of ZnO is generally attributed to point defects which introduce deep level traps inside band gap. The decreases in the peak intensity could be attributed to filling up of the deep level states by additional electrons from substitutional Ti atoms in the lattice or at the surface. Later, we discuss in detail whether Ti is actually involved in ZnO NPs via X-ray photoemission spectroscopy (XPS). Same tendency was observed in digital images of the NPs dispersed in less and nonpolar solvents such as isopropanol and chlorobenzene (Fig. S2).

We also checked colloidal stability of pZnO solution with addition of acetylacetonate (acac) ligands only, to confirm whether Ti atoms can help acac ligands adhere to the surface of ZnO NPs or not. The acac ligands were added in the same amounts as fZnO NPs. As shown in Fig. S3, similar to pZnO NPs, poor colloidal stability was observed in pZnO NPs with acac ligands; all solutions showed milky suspension two weeks later, which originated from aggregation of NPs. This indicates that the acac ligands alone cannot be attached to the surface of pZnO NPs. Therefore, this can be one of evidences that Ti atoms are necessary to attach acac ligands on the surface of ZnO NPs, which will be discussed subsequently.

**Figure 2:**
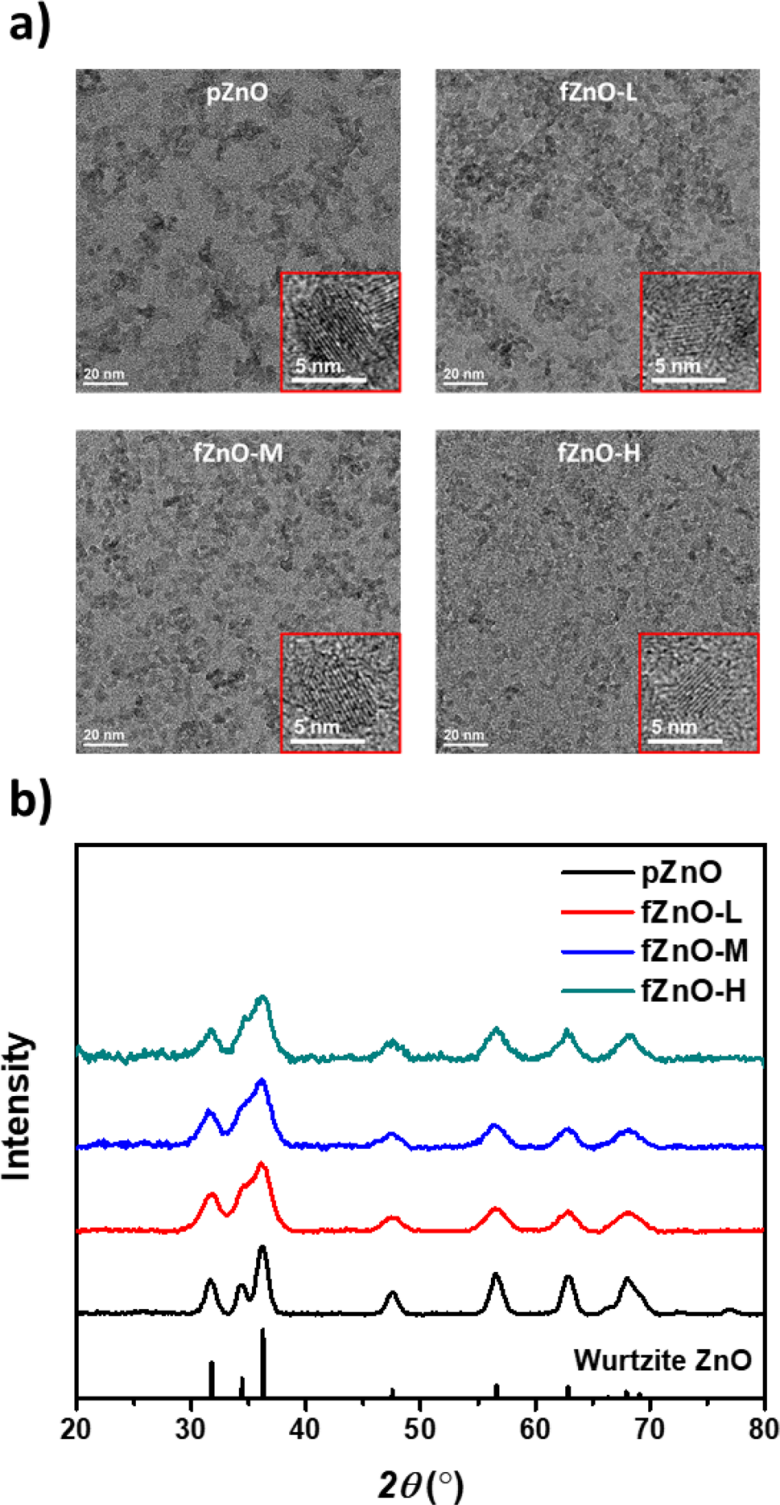
(**a**) Transmission electron microscope images and (**b**) X-ray diffraction patterns of fZnO NPs (Inset: magnified images of individual nanoparticles).

**Figure 3:**
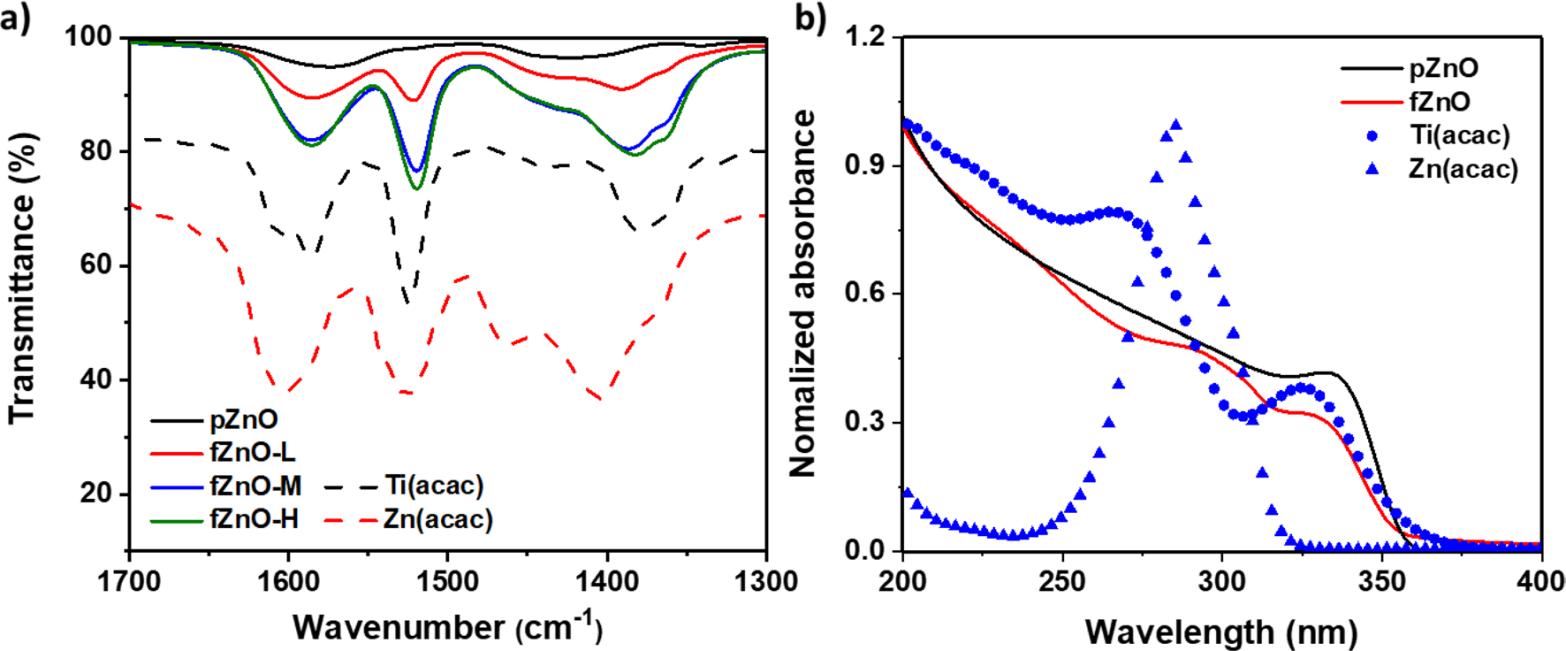
(**a**) IR spectra of fZnO NPs. Dashed line exhibits the spectrum of Zn(acac) and Ti(acac). (**b**) UV–Vis absorption spectra of pZnO, fZnO, Zn(acac), and Ti(acac) at UV region.

The influence of Ti(acac) on ZnO NPs at the atomic scale was investigated via transmission electron microscope (TEM), as shown in Fig. 2a. For pZnO, it was observed that dozens of spherical NPs formed huge agglomerates. While, the addition of Ti(acac) triggered to reduce the degree of aggregation and each NP was homogeneously distributed throughout the images for fZnOs. Magnified images, shown as inset in each image, reveals that the size and shape of NPs were maintained regardless of the Ti(acac) concentration. Figure 2b represents the X-ray diffraction (XRD) patterns of ZnO NPs. Strong and well-defined diffraction patterns of reference NPs at 2*θ* = 31.8°, 34.4°, 36.3°, 47.5°, 56.6°, 62.9°, and 68.0° correspond to (100), (002), (101), (102), (110), (103), and, (112) of Wurtzite ZnO, respectively. As increasing the concentration of Ti(acac), the peaks became weaker and broader, which were commonly observed in nanostructures compared to its bulk counterpart^29^. In the XRD data, broad and weak peak were observed due to small size of NPs or amorphous particle. The size and crystallinity of NPs were maintained regardless of the concentration of Ti(acac) as shown in Fig. 3a. However, the degree of aggregation was reduced in proportional to Ti(acac) concentration and each NP was homogeneously distributed. It is evident that the broad and weak peak was due to the decrease of aggregation. While, the peaks were observed at same positions, regardless of Ti(acac) treatment. In Fig. S4, reduced surface RMS roughness of fZnO thin films to less than a half (2.10, 1.54, and 1.26 nm for fZnO-L, fZnO-M, and fZnO-H, respectively), compared to pZnO thin film (4.01 nm) further proves the effectiveness of Ti(acac) to prevent NPs from interparticle attraction.

### Surface and elemental analysis of functionalized nanoparticles

IR spectroscopy is an efficient tool to characterize the organic-functionalized surface of inorganic NPs. In order to identify molecular interactions between Ti(acac) and the surface of ZnO NPs, IR spectra of pZnO, fZnO-L, M, and H are collected and compared in Fig. 3a. Two weak vibrational bands of pZnO were observed at 1574 cm^−1^, 1423 cm^−1^, which is attributed to the residual acetate ligands on the surface of pZnO. For three fZnOs, strong vibrational bands were observed at 1586, 1520, and 1437 cm^−1^, which correspond to the C =O and C=C vibrational bands of zinc acetylacetonate (Zn(acac))^30^, with a slight spectral shift to lower wavenumbers. The shift can be caused by lengthened bonds of Zn–O at the surface of the NPs compared to tightly bound metal–ligand bond in Zn(acac) complex^30,31^. Nearly identical IR intensities of fZnO-M and H at three bands reveals that the amount of acetylacetonates on the surface of the NPs is likely to be saturated after 41 μM of Ti(acac) addition. It is found that fZnOs exhibit reduced surface hydroxyl groups with broad vibrational band around 3400 cm^-1^, as increasing the concentration of Ti(acac) (Fig. S5). The strong correlation between surface RMS roughness and IR intensity at three featured vibrational bands (Fig. S6) supports our statement that reduced NP aggregation is mainly due to surface functionalization of ZnO NPs by Ti(acac). It is noteworthy that most metal acetylacetonate complexes exhibit similar IR responses^32,33^, which may give rise to the concerns that the collected vibrational bands arise from Ti(acac) rather than new Zn-(acac) bonds formed at the surface of the NPs. Yet, strong doublet peaks around 1600 cm^−1^, which is a distinct feature of Ti(acac), were not observed in Fig. 3a. This suggests that Ti(acac) is dissolved in methanol in the form of Ti^4+^ and bidentate (acac)^−^ ligands, which is, then, attached to the surface of the NPs. The dissociation of Ti(acac) and the formation of Zn(acac) in fZnOs were further confirmed in Fig. 3b. Two strong absorption peaks of Ti(acac) at 270.5 and 324.5 nm are not shown in fZnO spectrum. Rather, its strong absorption at 285 nm corresponds to distinct absorption feature of Zn(acac). The feature from Zn(acac) explains strong light absorption of fZnOs in the wavelengths below 320 nm, shown in Fig. 1b. This result is consistent with the discussion on Fig. 3a. X-ray photoelectron spectroscopy (XPS) was carried out to characterize chemical composition of the processed ZnO thin films. O1s spectra in Fig. S7 exhibit two peaks at 531.5 and 530.0 eV. The former corresponds to surface hydroxyl group of ZnO and the latter to Zn–O bond in the lattice^34^. The gradual increase in the intensities at 531.5 eV was observed as increasing the concentration of Ti(acac), which possibly be interpreted as a result of increased number of surface OH^-^ groups. However, it is controversial to the observation of reduced OH groups in fZnO samples as shown in Fig. S5. Another possible explanation is based on the fact that Ti–O bond in Ti doped ZnO is also observed at 531.5 eV^35,36^. It is found that Ti atoms are involved in the thin film as two sharp peaks at 464.0 and 458.3 eV, which corresponds to Ti 2p_1/2_ and 2p_3/2_ peaks in oxide lattice. Moreover, a slight shift of peaks to higher binding energy in Zn 2p spectra is possibly due to the de-shielding of positive charge of Zn nucleus caused by strong attractive force of substitutional Ti atoms to Zn core electrons, resulting in higher binding energy. From XPS results, it is found that Ti atoms are involved in the ZnO thin film and affect to the electronic structure of ZnO thin film, which is also confirmed via reduced PL intensities in Fig. 1c. Yet, it is not certain whether Ti is actually positioned at the surface of the NPs or inside the oxide lattice. A further study with more focus on it is therefore required.

### Device applications

**Figure 4:**
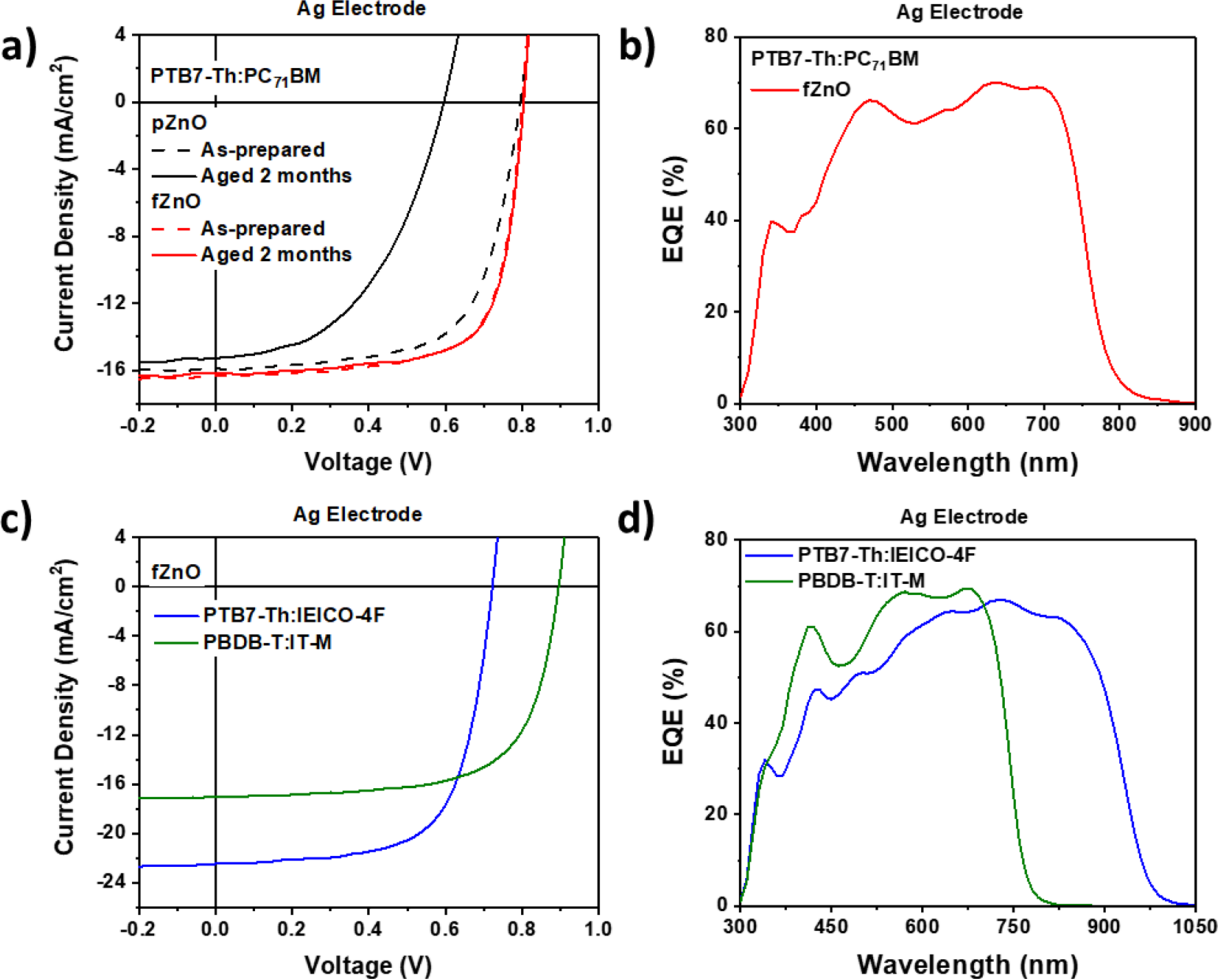
(**a**) *J-V* characteristics and (**b**) EQE spectra of PTB7-Th:PC_71_BM based PSCs using p- and fZnO NPs as ETLs comparing with as-prepared and aged for 2 months of each NPs. (**c**) *J–V* characteristics and (**d**) EQE spectra of PTB7-Th:IEICO-4F and PBDB-T:IT-M based PSCs with fZnO NPs as ETLs.

We fabricated PTB7-Th:PC_71_BM based bulk heterojunction solar cells using p- and fZnOs. The device structure comprises of indium tin oxide (ITO)/ poly(3,4-ethylenedioxythiophene):poly(styrenesulfonate) (PEDOT:PSS)/PTB7-Th:PC_71_BM/ZnO/Ag. pZnO and fZnO NPs were dispersed in methanol (MeOH) with concentration of 10 mg/ml and 5 mg/ml, respectively, which is optimum condition. Ag was intentionally used as top metal electrode to introduce energy level mismatch in the device where electron transfer from the active layer to Ag is energetically not favorable. The mismatch is corrected only when ZnO works as electron transport layer properly. Current density–voltage (*J-V)* characteristics of the PTB7-Th:PC_71_BM-based device with p- and fZnO are shown in Fig. 4a, respectively. Photovoltaic parameters of the devices are summarized in Table 1. Compared to pZnO devices, fZnO devices exhibited higher performance, especially in fill factor (FF*)* (0.66 → 0.71). Due to high colloidal stability of fZnOs, uniform thin film can be formed on the active layer leading to more efficient charge transport, compared to pZnOs. The significant deterioration of device performance was observed with aged pZnOs, resulting in decreased PCE by 47.5%. Especially open-circuit voltage (*V*_OC_) and FF were reduced dramatically, from 0.80 to 0.60 V and from 0.66 to 0.48, respectively. This is originated from energetically mismatch between active layer and Ag electrodes; due to poor film coverage of aged pZnOs, direct contact can be induced partially. On the other hand, aged fZnO devices exhibited almost similar *J*-*V* characteristics with those of as-prepared fZnO devices, as shown in Fig. 4a. This indicates that the colloidal stability and quality of fZnOs were maintained successfully even aging for 2 months under ambient condition. Efficient electron transporting ability of fZnOs was further demonstrated by fabricating PSCs using two polymeric blends, PTB7-Th: 2,2′-[[4,4,9,9-Tetrakis(4-hexylphenyl)-4,9-dihydro-s-indaceno[1,2-b:5,6-b']dithiophene-2,7-diyl]bis[[4-[(2-ethylhexyl)oxy]-5,2-thiophenediyl]methylidyne(5,6-difluoro-3-oxo-1H-indene-2,1(3H)-diylidene)]]bis[propanedinitrile] (IEICO-4F)^37^ and poly[(2,6-(4,8-bis(5-(2-ethylhexyl)thiophen-2-yl)-benzo[1,2-b:4,5-b’]dithiophene))-alt-(5,5-(1′,3′-di-2-thienyl-5′,7′-bis(2-ethylhexyl)benzo[1′,2′-c:4′,5′-c’]dithiophene-4,8-dione)] (PBDB-T): 3,9-bis(2-methylene-((3-(1,1-dicyanomethylene)-6/7-methyl)-indanone))-5,5,11,11-tetrakis(4-hexylphenyl)-dithieno[2,3-d:2′,3′-d’]-s-indaceno[1,2-b:5,6-b’]dithiophene (IT-M)^38^. The former blend, PTB7-Th:IEICO-4F, exhibited high *J*_SC_, and the latter blends, PBDB-T:IT-M, exhibits high *V*_OC_. Here, we used Al as top electrodes instead of Ag for the better energy level alignment in conventional structure. The *J–V* characteristics of PTB7-Th:IEICO-4F and PBDB-T:IT-M based PSCs are shown in Fig. 4c, and the corresponding photovoltaic parameters are summarized in Table 2. PTB7-Th:IEICO-4F blend devices exhibited strong dependence on the presence of ZnO layer. The averaged PCEs were greatly improved by ~ 2 times (from 5.23 to 10.6%) with use of fZnO layer, compared to the control devices (without fZnOs). Especially *V*_OC_ and FF were greatly improved from 0.53 to 0.72 V, and from 0.51 to 0.67, respectively, indicating fZnO layer works well as ETL for PTB7-Th:IEICO-4F blend devices. Meanwhile, PBDB-T:IT-M devices exhibited less dependence on ZnO layer compared to PTB7-Th:IEICO-4F based devices, but still considerable performance enhancement was observed. The averaged PCEs for the devices with fZnOs were improved by 1.3 times (from 7.32 to 9.90%), compared to the control devices, and the best PCE was 10.3%. These results indicate that the fZnO layer works as ETL effectively for fullerene and non-fullerene organic solar cells. External quantum efficiency (EQE) spectra of the corresponding devices are shown in Fig. 4b,d. Calculated *J*_SC_s from EQE spectra of each device are well consistent with each *J*-*V* characteristics.

**Table 1:** Summary of photovoltaic parameters of PTB7-Th:PC_71_BM based polymer solar cells.

ETL	Aging	*J*_SC_ (mA cm^-2^)	*V*_OC_ (V)	*FF*	PCE (%)
pZnO	As-prepared	15.9 (15.7 ± 0.38)	0.80 (0.80 ± 0.00)	0.66 (0.65 ± 0.02)	8.32 (8.15 ± 0.22)
	2 months	15.3 (15.2 ± 0.25)	0.60 (0.58 ± 0.01)	0.48 (0.47 ± 0.01)	4.37 (4.14 ± 0.14)
fZnO	As-prepared	16.3 (15.9 ± 0.31)	0.80 (0.80 ± 0.00)	0.71 (0.71 ± 0.01)	9.32 (9.07 ± 0.16)
	2 months	16.2 (15.2 ± 0.53)	0.80 (0.80 ± 0.00)	0.72 (0.71 ± 0.01)	9.32 (8.72 ± 0.30)

**Table 2:** Summary of photovoltaic parameters for PTB7:IEICO-4F and PBDB-T:IT-M based polymer solar cells.

Active layer	ETL	*J*_SC_ (mA cm^-2^)	*V*_OC_ (V)	*FF*	PCE (%)
PTB7-Th:IEICO-4F	X	20.7 (20.0 ± 0.43)	0.53 (0.52 ± 0.01)	0.51 (0.50 ± 0.02)	5.66 (5.23 ± 0.38)
	fZnO	22.5 (22.2 ± 0.34)	0.72 (0.72 ± 0.00)	0.67 (0.66 ± 0.00)	10.8 (10.6 ± 0.17)
PBDB-T:IT-M	X	16.5 (16.2 ± 0.21)	0.85 (0.85 ± 0.00)	0.54 (0.53 ± 0.01)	7.53 (7.32 ± 0.17)
	fZnO	17.0 (16.6 ± 0.37)	0.90 (0.89 ± 0.00)	0.67 (0.67 ± 0.01)	10.3 (9.90 ± 0.27)

**Figure 5:**
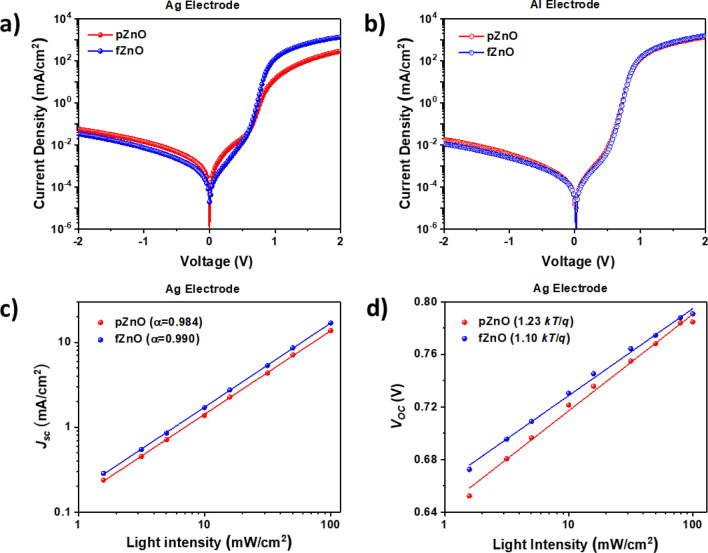
*J-V* characteristics in the dark PTB7-Th:PC_71_BM based PSCs using (**a**) Ag electrode and (**b**) Al electrode for p- and fZnO as ETL. Light intensity dependence of (**c**) *J*_SC_ and (**d**) *V*_OC_ for PTB7-Th:PC_71_BM PSCs with Ag electrode devices.

We measured *J-V* curves in the dark using PTB7-Th:PC_71_BM blend devices with Ag and Al electrodes as shown in Fig. 5a,b, respectively, to compare the effects of p- and fZnOs as ETL. Series and shunt resistances (*R*_s_ and *R*_sh_, respectively) of each devices are summarized in Table S1. In case of Al electrode, its work function is well matched with lowest unoccupied molecular orbital (LUMO) level of acceptor materials in conventional OSCs. Therefore, diode characteristics in both p- and fZnO devices were almost similar whether ZnO NPs are fully covered or not on top of the active layer. In case of Ag electrode, however, its work function is not completely well matched with LUMO level of acceptor materials, so the device performance can be significantly affected by film quality and uniformity of ZnO NPs layer. Since pZnO NPs cannot be fully covered on top of the active layer due to their significant self-aggregation, poor diode characteristics were observed with high *R*_s_. On the other hand, fZnO NPs can be fully covered on top of active layer with uniform film quality, so the devices with fZnO showed excellent diode characteristics with low *R*_s_. These results are well consistent with the photovoltaic properties that the fZnO devices with Ag electrodes showed better performance compared to the pZnO devices. To investigate charge recombination properties, light intensity dependence on *J*_SC_ and *V*_OC_ were plotted with PTB7-Th:PC_71_BM PSCs as shown in Fig. 5c,d, respectively. The fitted curves of *J*_SC_ vs. light intensity followed the power-law equation expressed by

1 $$ J_{SC} \propto I^{\alpha } $$

where *I* is incident light intensity. If *α* is close to unity, it indicates that the system has weak bimolecular recombination^39,40^. For the devices with p- and fZnO, α values are 0.984 and 0.990, respectively. fZnO devices showed slightly higher *α* value, indicating that fZnO devices have weaker bimolecular recombination compared to those of pZnO. Weak bimolecular recombination could be attributed to excellent film coverage of fZnOs on top of active layer, which is originated from improved colloidal stability.

If bimolecular recombination is the only loss mechanism in that given system, the *V*_OC_ follows

2 $$ V_{OC} = \frac{{HOMO_{donor} - LUMO_{acceptor} }}{q} - \frac{kT}{q}\ln \left[ {\frac{{\left( {1 - P_{D} } \right)\gamma N_{C}^{2} }}{{P_{D} G}}} \right] $$

where HOMO_donor_ is highest occupied molecular orbital (HOMO) level of donor material, LUMO_acceptor_ is LUMO level of acceptor materials, *q* is the elementary charge, *k* is the Boltzmann constant, *T* is temperature in Kelvin, *P*_D_ is dissociation probability of exciton pair, *γ* is the Langevin recombination constant, *N*_C_ is the effective density of states, and *G* is the generation rate of bound excitons. From *V*_OC_ vs. ln(*I*) characteristics, if the slope value is high, there are significant trap-assisted (Shockley–Read–Hall, SRH) recombination^41–43^. In Fig. 5d, the slope for the device with p- and fZnO are 1.23 kT/q and 1.10 kT/q, respectively. The pZnO devices showed higher slope value than the fZnO devices, which indicates pZnO devices suffer from more significant trap-assisted recombination compared to fZnO devices. This implies that uniform and high-quality thin film of ZnO NPs can be achieved by functionalizing Ti(acac) to ZnO NPs, leading to excellent photovoltaic device performance with negligible bimolecular and trap-assisted recombination.

## Discussion

In conclusion, a coordination complex, Ti(acac), was employed as an efficient bifunctional stabilizer of ZnO NPs dispersed in methanol. The surfaces of the NPs were successfully functionalized with acac, which effectively reduced aggregations of the NPs. The suppression of agglomerates formation in the NP solutions led to smoother surface roughnesses of ZnO thin films. Tetravalent Ti atoms in ZnO lattice passivated deep level traps of the NPs, which led to significant reduction in green luminescence. It was also found that Ti(acac) exhibited its functionality even in other solvents of different polarities such as isopropanol and chlorobenzene. fZnO was then employed to the PSCs with three different polymeric blends. PTB7-Th:PC_71_BM devices using as-prepared and aged fZnO were compared and exhibited nearly identical *J-V* characteristics. The versatility of fZnO was further proved in two nonfullerene polymeric blends, PTB7-Th:IEICO-4F and PBDB-T:IT-M. We found that fZnO works as an efficient electron transport layer in both devices without deteriorating optimized polymeric blends morphology.

We demonstrated simple and universal method to improve colloidal stability of ZnO nanoparticles and successfully applied them to the PSCs with conventional structure, proving long-term stability of ZnO nanoparticle solution. Cheap, robust, and stable ZnO NPs with high colloidal stability will provide availability of conventional structure for highly stable and efficient PSCs, which have been mostly achieved in inverted structure so far. Furthermore, an availability of the method in a solvent with an opposite polarity provides their potential applications in perovskite solar cells, which is one of the hottest topics in recent photovoltaic research.

## Methods

### Materials

Zinc acetate dihydrate, Potassium hydroxide (KOH), methanol, ti(acac) and acac were purchased from Aldrich and used without further purification. PC_71_BM was purchased from Organic Semiconductor Materials (OSM, Republic of Korea). PTB7-Th, IEICO-4F, PBDB-T and IT-M were purchased from 1-Material.

### ZnO nanoparticle synthesis

ZnO NP solutions were prepared using the method reported elsewhere with a slight modification^22,23^. Briefly, Zinc acetate dihydrate (2.95 g, 13.4 mmol) was dissolved in 135 mL of methanol at 60 ℃. KOH solution (1.48 g, 26.7 mmol of KOH in 55 mL methanol) was added dropwise to the zinc acetate solution under vigorous stirring and kept at 60 ℃ for 2 h and 15 min. As-synthesized ZnO NPs were precipitated by centrifugation and washed twice using methanol. The precipitants were finally dispersed in methanol at high concentration as a stock solution (20 ~ 50 mg/mL). Before using it, the stock solution was diluted to the desired concentration (5 ~ 20 mg/mL). For the preparation of fZnO NP solutions, 20.5 μM, 41.0 μM, and 82.0 μM of Ti(acac) were added to the pZnO NP solution then sonicated, denoted as fZnO-L, M, and H.

### Material characterization

UV–vis absorption spectra were obtained in the wavelength range of 200 to 800 nm using an Agilent Cary 5000 UV–Vis-NIR spectrometer at room temperature. PL spectra were recorded between 350 to 800 nm wavelength using a Varian Cary Eclipse fluorescence spectrophotometer at room temperature. Highly diluted solutions were prepared in 1 cm path-length quartz cuvettes in order not to exceed the highest possible intensity measured in the instruments, mostly occurs at short wavelengths. XPS spectra were collected using a Thermo Fisher scientific ESCALAB250XI with a monochromated Al-Kα X-ray source at a base pressure of 1.0 × 10^−9^ Torr. The samples were prepared by spin-coating onto 80 nm gold films on Si substrates with room condition. XRD measurement was carried out using a Bruker AXS D8 Advance X-ray Diffractometer with CuKα radiation. The samples were examined in the range of 20 to 80° by 0.02° (2*θ*) and the scan rate is 2.4° min^−1^. AFM height and phase images were taken with a Veeco DI-3100 AFM microscope in the tapping mode. The samples were prepared by spin-coating on ITO substrate. FT-IR absorption spectra were recorded on Varian 670 / 620 FT-IR Microscopes in ATR mode. The samples were diluted in MeOH before the measurement. The TEM images were collected by JEOL JEM-2100F Field Emission Electron Microscope. The samples were prepared by a drop-dry method on carbon-coated copper grids.

### Device fabrication and characterization

Polymer solar cells were fabricated with conventional structure. ITO patterned glass substrates were sequentially cleaned with distilled water, acetone, and isopropanol by ultrasonication for 10 min. The substrates were dried at 100 ℃ overnight. PEDOT:PSS was spin-coated onto the cleaned ITO substrate and annealed at 140 °C for 10 min.

For PTB7-Th:PC_71_BM based devices, the mixture of donor and acceptor with a 1:1.5 weight ratio (11 mg/ml) were dissolved in chlorobenzene (CB):diphenyl ether (DPE) (97:3 v/v). Two non-fullerene blend solutions were prepared with a 1:1.25 weight ratio (12 mg/ml) dissolved in CB:1-chloronaphthalene (CN) (98:2 v/v) and 1:1 weight ratio (11 mg/ml) in CB:1,8-diiodooctane (DIO) (99.5:0.5 v/v) for PTB7-Th:IEICO-4F and PBDB-T:IT-M, respectively. The solutions were magnetically stirred for 3 h at 60 ℃. The polymeric blend solutions were spin-cast onto PEDOT:PSS layer in a nitrogen (N_2_)-filled glove box. Especially, PBDB-T:IT-M layer was annealed at 100 ℃ for 10 min. after spin-cast. ZnO NP solutions were spin-coated on the photoactive layer. Subsequently, the device was pumped down under vacuum (< 10^–6^ Torr), and Ag (85 nm, for PTB7-Th:PC_71_BM) and Al (100 nm, for PTB7-Th:IEICO-4F and PBDB-T:IT-M) were deposited by thermal evaporation. *J*−*V* characteristics of the devices were obtained using a Keithley 2635A source measure unit in the N_2_ filled glove box. A mask (3.51 mm^2^) made of a thin metal was attached to each cell before characterization under AM 1.5G illumination at 100 mW cm^−2^. EQE of the devices were determined using a PV Measurements QE system using monochromatic light from a xenon lamp under ambient conditions. The monochromatic light was chopped at 100 Hz, and the intensity was calibrated relative to a standard Si photodiode.

## Supplementary information

Supplementary Information
