# Supplementary material for: High colloidal stability ZnO nanoparticles independent on solvent polarity and their application in polymer solar cells
Source: Sci Rep. 2020 Oct 22;10:18055. doi: 10.1038/s41598-020-75070-0 (PMC7582139; doi:10.1038/s41598-020-75070-0)
Supplement: Supplementary file 1 — Supplementary Information [file 41598_2020_75070_MOESM1_ESM.docx]

Supporting Information

High Colloidal Stability ZnO Nanoparticles Independent on Solvent Polarity and Their Application in Polymer Solar Cells

**Woojin Lee^1,†^, Jiwoo Yeop^1,†^, Jungwoo Heo^2,†^, Yung Jin Yoon^1^, Song Yi Park^1^, Jaeki Jeong^1^, Yun Seop Shin^1^, Jae Won Kim^1^, Na Gyeong An^1^, Dong Suk Kim^3^, Jongnam Park^1,^*, and Jin Young Kim^1,2,^***

^1^Department of Energy Engineering, Ulsan National Institute of Science and Technology (UNIST), 50 UNIST-gil, Ulju-gun, Ulsan 44919, Republic of Korea

^2^Department of Physics, Ulsan National Institute of Science and Technology (UNIST), 50 UNIST-gil, Ulju-gun, Ulsan 44919, Republic of Korea.

^3^KIER-UNIST Advanced Center for Energy, Korea Institute of Energy Research (KIER), UNIST-Gil 50, Eonyang-eup, Ulju-gun, Ulsan 689-851, Republic of Korea

*jykim@unist.ac.kr (Prof. J. Y. Kim)

*jnpark@unist.ac.kr (Prof. J. Park)

^†^These authors contributed equally to this work

**
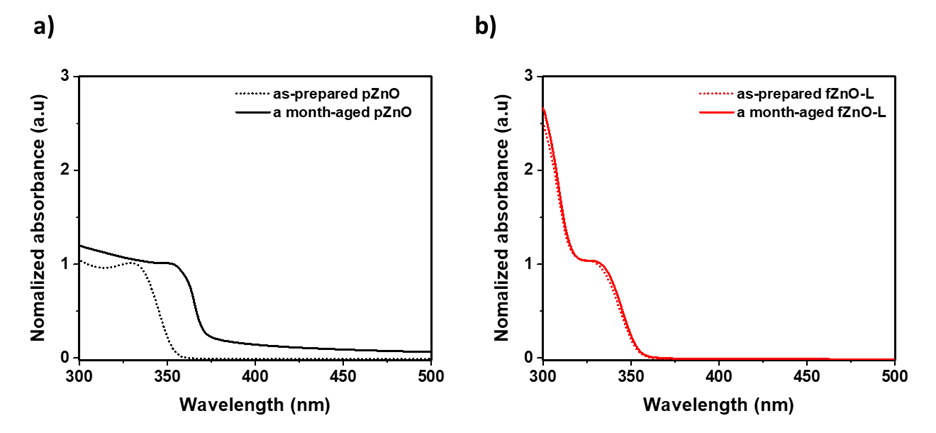
Figure S1.** Comparison of UV-vis absorption spectra of **a)** p- and **b)** fZnO under ambient conditions (25℃ and 50-70% RH) for a month.

*
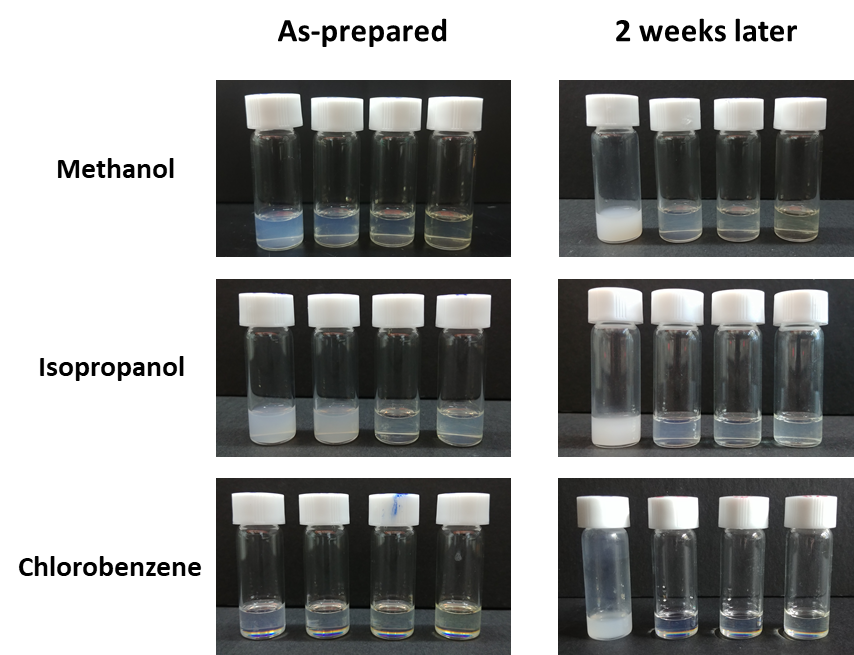
*

**Figure S2.** Colloidal stability of pZnO, fZnO-L, fZnO-M, and fZnO-H (from left to right, in turn) in a variety of solvents


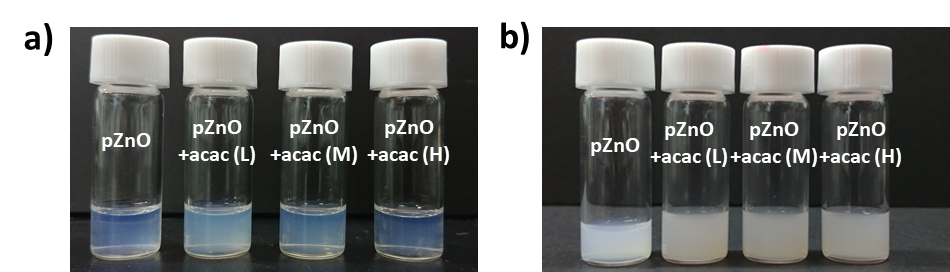


**Figure S3.** Photographs of acac functionalized ZnO NP solutions, denoted as pZnO, pZnO+acac (L), pZnO+acac (M) and pZnO+acac (H) (none, low, mid and high concentration of acac). **a)** As-prepared and **b)** 2 weeks later.

*
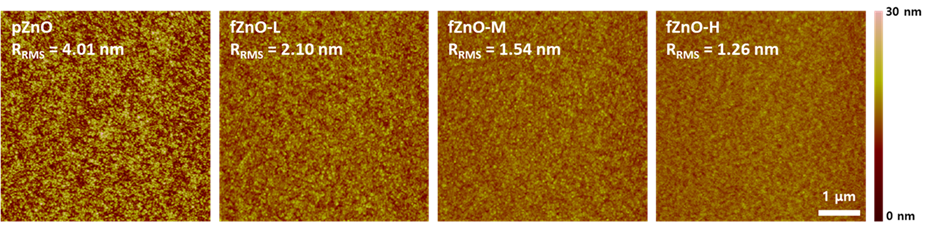
*

**Figure S4.** Atomic force microscope topographic images of ZnO thin films prepared on ITO substrates

**
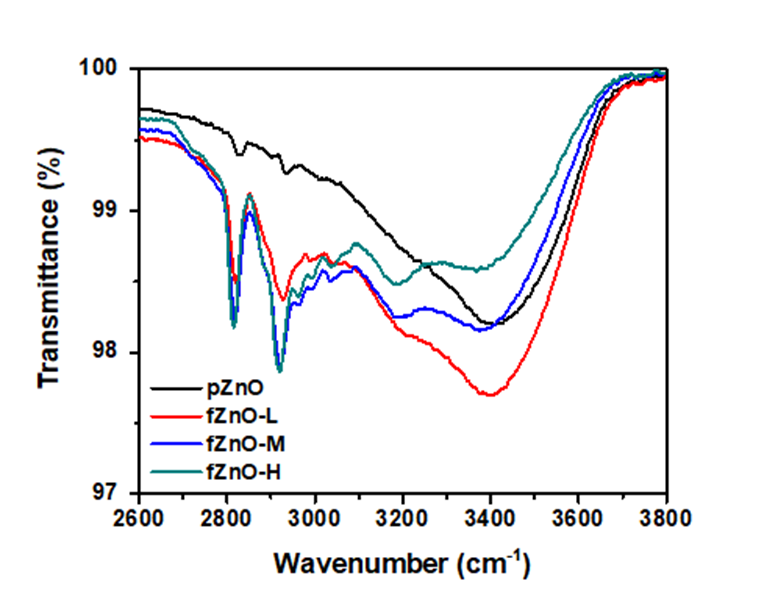
**

**Figure S5.** FT-IR spectra of ZnO NPs in the range between 2600 and 3800 cm^-1^


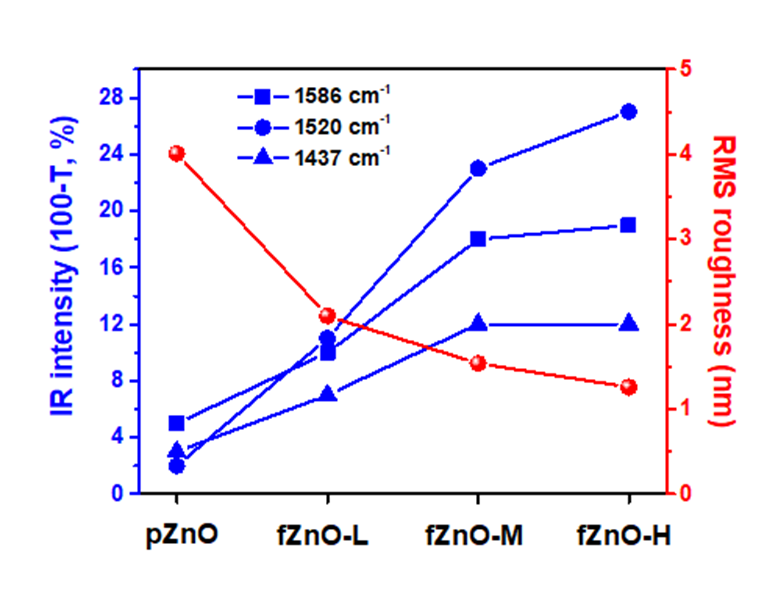


**Figure S6.** The relation between IR intensities and RMS roughness ZnO NPs


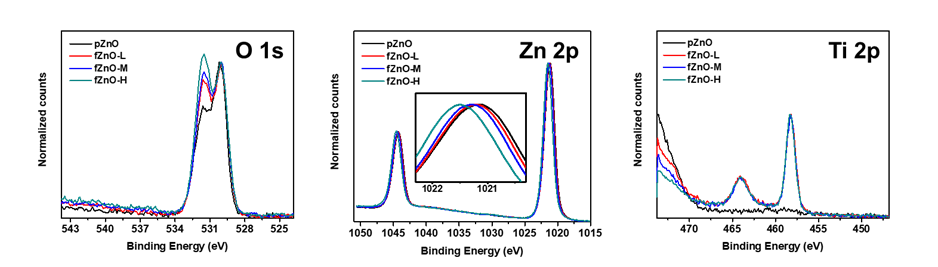


**Figure S7.** X-ray photoelectron spectra of ZnO NP thin films prepared on Au coated silicon substrate

| ETL | *R*_sh_  [kΩ·cm^2^] | *R*_s_  [Ω·cm^2^] |
| --- | --- | --- |
| pZnO | 121 | 2.15 |
| fZnO | 233 | 0.65 |

**Table S1.** Summary of *R*_sh_ and *R*_s_ for PTB7-Th:PC_71_BM based polymer at Ag electrode.
